# PDGF-BB overexpressing dental pulp stem cells improve angiogenesis in dental pulp regeneration

**DOI:** 10.3389/fbioe.2025.1578410

**Published:** 2025-04-24

**Authors:** Wentao Jiang, Shuhan Duan, Weiping Li, Huijiao Yan, Chenli Si, Ningwei Xu, Yishuai Li, Wenjie Zhang, Shensheng Gu

**Affiliations:** 1 Department of Endodontics, Shanghai Ninth People’s Hospital, Shanghai Jiao Tong University School of Medicine, Shanghai, China; 2 College of Stomatology, Shanghai Jiao Tong University, Shanghai, China; 3 National Center for Stomatology and National Clinical Research Center for Oral Diseases, Shanghai, China; 4 Shanghai Key Laboratory of Stomatology and Shanghai Research Institute of Stomatology, Shanghai, China; 5 Department of Oral Surgery, Shanghai Ninth People’s Hospital, Shanghai Jiao Tong University School of Medicine, Shanghai, China; 6 The Affiliated Stomatological Hospital of Nanjing Medical University, State Key Laboratory Cultivation Base of Research, Prevention and Treatment for Oral Diseases, Jiangsu Province Engineering Research Center of Stomatological Translational Medicine, Nanjing, China; 7 Department of Prosthodontics, Shanghai Ninth People’s Hospital, Shanghai Jiao Tong University School of Medicine, Shanghai, China; 8 Department of Dermatology, Shanghai Ninth People’s Hospital, Shanghai Jiao Tong University School of Medicine, Shanghai, China

**Keywords:** dental pulp stem cells, vascularization, single-cell RNA sequencing, endothelial, dental pulp regeneration

## Abstract

**Introduction:**

Angiogenesis represents a critical challenge in dental pulp regeneration due to the tissue’s restricted nutrient supply through a 0.5-mm apical foramen. While dental pulp stem cells (DPSCs) hold regenerative potential, their limited vascularization capacity impedes clinical applications. Through Single-cell RNA sequencing (scRNA-seq) analysis of human dental pulp, we discovered a PDGF (+) mesenchymal subset exhibiting enhanced angiogenic signatures, suggesting targeted cell selection could overcome this bottleneck.

**Methods:**

ScRNA-seq identified PDGF (+) subpopulation in human pulp samples, validated through multiplex immunohistochemical of the localization of PDGF/CD73/CD31. PDGF-BB-overexpressing DPSCs were engineered via lentiviral vectors. Functional assessments included: 1) CCK-8/Edu/cell cycle/transwell assays for proliferation and migration ability 2) HUVECs co-culture models analyzing chemotaxis and tube formation 3) Vascularized tissue formation in rat kidney capsule transplants.

**Results and Discussion:**

The CD73 (+) PDGF (+) subpopulation demonstrated spatial correlation with CD31 (+) vasculature. PDGF-BB overexpression enhanced DPSCs' proliferative capacity and migration capacity. Co-cultured HUVECs exhibited increased tube formation with PDGF-BB group. *In vivo* transplants generated more vascular structures containing CD31 (+) endothelia. These findings establish PDGF-BB engineering as an effective strategy to amplify DPSCs' angiogenic potential, while emphasizing the therapeutic value of functionally-defined stem cell subpopulations in pulp regeneration.

## 1 Introduction

Root canal treatment is currently the prior method for the clinical treatment of irreversible pulpitis (Connert et al., 2022). However, for teeth that have undergone root canal treatment, due to the lack of dental pulp tissue, they no longer possess functions such as immune response, the ability to perceive external stimuli, and the formation of secondary dentin. Additionally, these teeth face an increased risk of root fracture, which can easily lead to treatment failure (Eramo et al., 2018; Xie et al., 2021). Therefore, regenerating functional dental pulp tissue is essential for the long-term preservation of teeth. With recent advancements in regenerative medicine, dental pulp regeneration using tissue engineering techniques has become a key area of research (Li et al., 2024; Widbiller and Galler, 2023).

The discovery of dental pulp stem cells (DPSCs) in 2000 marked a significant milestone in stem-cell-based dental pulp regeneration research (Gronthos et al., 2000). Originating from neural crest cells within the early ectodermal tissues of the craniofacial region (Achilleos and Trainor, 2012), DPSCs possess distinctive biological characteristics, including exceptional self-renewal capabilities and multipotent differentiation potential (Sui et al., 2020). These unique properties have established DPSCs as a promising candidate for various applications in regenerative medicine, particularly in dental tissue engineering and repair (Janebodin et al., 2011; Cabaña-Muñoz et al., 2023). However, despite their remarkable regenerative potential, the clinical application of DPSCs faces significant challenges, particularly in achieving satisfactory therapeutic outcomes through direct transplantation (Dissanayaka and Zhang, 2017). The most prominent limitation lies in the inadequate vascularization of transplanted DPSCs, which severely compromises their survival and functional integration within host tissues (Huang et al., 2020). This insufficient vascular network formation not only leads to poor nutrient and oxygen supply but also results in limited waste removal, ultimately causing substantial cell death and impaired tissue regeneration (Rouwkema and Ali, 2016). Moreover, during *in vitro* culture and expansion, DPSCs are prone to senescence, resulting in a decline in their proliferative and differentiation capabilities (Morsczeck, 2019; Shi et al., 2024). Additionally, DPSCs exhibit heterogeneity, comprising cells with varying properties and functions. This variability makes it challenging to precisely regulate their behavior and differentiation pathways (Kok et al., 2022; Nel et al., 2022).

In response to these limitations, researchers have developed multiple innovative strategies to enhance the vascularization capacity and tissue repair potential of DPSCs. Three primary approaches have emerged as particularly promising: (1) co-transplantation with endothelial progenitor cells to facilitate vascular network formation (Dissanayaka et al., 2012; Khayat et al., 2017); (2) the design of advanced bioactive scaffolds incorporating controlled growth factor delivery systems (Han et al., 2024; Li et al., 2016); and (3) genetic engineering techniques to upregulate key angiogenic factors (Zhu et al., 2019). Among these strategies, genetic modification has shown remarkable potential by enabling precise manipulation of cellular functions. However, the clinical application of genetic modification approaches faces significant challenges. The introduction of exogenous genes or the overexpression of non-native genes in mesenchymal stem cells (MSCs) may lead to genomic instability, increasing the risk of tumorigenesis and resulting in unpredictable therapeutic outcomes (Shomali et al., 2020). Therefore, researchers have proposed a more refined strategy that focuses on identifying and characterizing native stem cell subpopulations involved in physiological angiogenesis within dental pulp tissue. This approach involves systematic screening for cells exhibiting intrinsic high angiogenic potential and their associated signaling pathways, followed by targeted expansion of these selected cells through controlled *in vitro* genetic modification to enhance pro-angiogenic signal expression.

The advent of single-cell RNA sequencing (scRNA-seq) and spatial transcriptomics technologies has revolutionized our understanding of cellular heterogeneity within dental pulp tissue. Zhao’s group (Ren et al., 2022) conducted a single-cell analysis of dental pulp tissue and identified that BMP receptor-enriched DPSCs may be involved in odontoblast differentiation and dentin mineralization. Wu’s group Di et al. (2024a) using cell communication analysis, identified a unique FN1^+^ DPSCs subpopulation with significant angiogenic potential. However, despite these advancements, a substantial proportion of DPSCs subpopulations remain poorly characterized, with their specific roles in pulp regeneration largely unexplored.

To overcome these limitations and explore the therapeutic potential of uncharacterized DPSCs subpopulations, we implemented a comprehensive research strategy. Initially, we utilized scRNA-seq to identify and characterize a unique mesenchymal stem cell subpopulation characterized by specific PDGF gene expression. CellChat analysis revealed that this PDGF-expressing subpopulation demonstrates enhanced intercellular communication with endothelial cells, suggesting its potential role in physiological angiogenesis during dental pulp development. Among the PDGF family members, PDGF-BB exhibits distinct biological superiority in promoting angiogenesis, particularly in oral hard and soft tissue regeneration (Galarraga-Vinueza et al., 2024). Unlike PDGF-AA (which binds exclusively to PDGFR-α) or PDGF-AB (with intermediate affinity), PDGF-BB demonstrates high-affinity binding to both PDGFR-α and PDGFR-β, enabling broader activation of downstream signaling cascades critical for vascular growth (Ch and Westermark, 1999; Spencer et al., 2017). This unique receptor interaction enhances endothelial cell proliferation, migration, and pericyte recruitment—key steps in stabilizing nascent microvasculature (Gianni-Barrera et al., 2018; Kemp et al., 2020). Building upon these findings, we established an *in vitro* model of PDGF-BB gene-modified DPSCs using lentiviral vector-mediated gene delivery. This approach enabled us to systematically investigate the effects of PDGF-BB overexpression on DPSC proliferation and migratory capacity. To further elucidate the angiogenic potential of these modified cells, we developed a co-culture system incorporating DPSCs and human umbilical vein endothelial cells (HUVECs). Our results demonstrated that PDGF-BB-overexpressing DPSCs exhibit significantly enhanced capabilities in endothelial cell recruitment and tube formation, key processes in vascular network development. Finally, through an *in vivo* rat kidney capsule transplantation model, we further validated that DPSCs genetically overexpressing PDGF-BB exhibit a stronger ability to form vascularized dental pulp tissue. Our findings provide important insights for advancing dental pulp regeneration therapy, highlighting the potential therapeutic significance of targeting specific DPSCs subsets to optimize dental pulp angiogenic regeneration.

## 2 Materials and methods

### 2.1 Single-cell RNA sequence data analysis

The single-cell RNA-seq data for this study were obtained from GSE164157 (Pagella et al., 2021). Raw sequence reads in FASTQ format from five samples of human dental pulp tissue were processed and aligned to theGRCh38 human reference transcriptome using the Cellrangerv7.1.0 pipeline with default parameters. The resulting gene expression matrices merged together using the Seurat software package version 5, followed by further cell filtration, normalization, clustering, and Uniform Manifold Approximation and Projection (UMAP) dimensional reduction. For each cell type, we rerun the Seurat cluster workflow to identify cell sub-types. Differentially expressed genes (DEGs) between the two subsets were identified using the FindMarkers function in the Seurat package. Subsequently, Gene Ontology (GO) analysis was performed using the “clusterProfiler” R package to investigate the potential functions of these DEGs. We utilize UCell (version 2.3.1) to score the angiogenesis-associated genes (Qing et al., 2022) expression for the two subcellular populations,which is based on the Mann-Whitney U statistic to evaluate gene feature scores. The cell-cell interactions and inference between different cell types were evaluated using R package CellChat v2.

### 2.2 Multiplex immunohistochemistry (mIHC) staining

The human dental pulp tissues obtained clinically were dehydrated and embedded in paraffin, and then 4 μm thick sections were prepared. The sections were first incubated in a drying oven at 60°C for 1 h and then immersed in xylene for 15 min and repeated 3 times for deparaffinization. Rehydration was carried out by sequentially immersing the slides in 100% ethanol, 95% ethanol, and 70% ethanol. Subsequently, the sections were placed in Tris-EDTA buffer (pH = 9.0) and heated in a high-power microwave for 5 min, followed by an additional 10-min heating at low power for antigen retrieval. After the slides cooled to room temperature, they were immersed in 3% hydrogen peroxide for 5 min to block endogenous peroxidase activity and then blocked with 3% BSA for 30 min. Thereafter, staining was performed using the mIHC multiplex fluorescent immunohistochemistry kit (WASci, WAS1504). The slides were incubated with the primary antibodies at 4 °C overnight. Then, the slides were washed three times in 1×PBS and incubated with the horseradish peroxidase (HRP)-conjugated secondary antibodies at room temperature for 50 min. After a quick wash in 1×PBS, they were incubated with the appropriate fluorophore-conjugated tyramide signal amplification (TSA) at room temperature for 10 min. Once again, the slides were exposed to microwave treatment to strip the tissue-bound primary/secondary antibody complexes in preparation for labeling the next primary antibody. The process of using primary antibodies (CD31, Abcam, ab281583; CD73, Proteintech, 12231-1-ap; anti-PDGFB, Abcam, ab23914), HRP-conjugated secondary antibodies (WASci, WAS1201), and fluorophore-conjugated TSA (WASci, WAS1003; WASci, WAS1004; WASci, WAS1006) was repeated until all markers were labeled. Finally, DAPI (WASci, WAS1301) was added to the slides, and they were incubated in the dark at room temperature for 10 min. The tissue samples were imaged using a PANNORAMIC MIDI II.

### 2.3 Cell isolation and culture

All procedures were approved by the Ethics Committee of Shanghai Ninth People’s Hospital Affiliated to Shanghai Jiao Tong University School of Medicine (SH9H-2021-T112-1). Primary human DPSCs were extracted from human dental pulp tissue. Specifically, healthy decay-free impacted wisdom teeth were collected and stored in a preservation solution of basal culture medium with 1% penicillin/streptomycin at 4°C. Informed consent was obtained from each patient (ranging from 18 to 25 years old). Pulp tissue was removed in the ultra-clean table, thoroughly rinsed with sterile PBS, and subsequently minced into small fragments of approximately 1–2 mm^3^. These minced tissue pieces were then plated in a petri dish and cultured in Dulbecco’s modified Eagle’s medium (DMEM; Gibco, United States) containing 20% fetal bovine serum (FBS; Every Green, China) and 1% penicillin/streptomycin (Gibco, United States) at 37°C in 5% CO_2_. After primary cell passage, the culture medium was changed to one containing 10% FBS. Cells at passages P3-P6 were used in this study. DPSCs were identified via the cell surface antigens CD14, CD45, CD34,CD73,CD105 and CD90 using a flow cytometry assay as described previously (W. Li et al., 2021). The multipotent differentiation potential of these cells was assessed using osteogenic (Cyagen Biosciences, HUXXC-90021) and adipogenic induction kits (Cyagen Biosciences, HUXXC-90031). Upon reaching the determined time point, the samples were subjected to staining with alkaline phosphatase, alizarin red, and oil red O, following the procedures described previously (Fei et al., 2024).

HUVECs were obtained from National Collection of Authenticated Cell Cultures, and cultivated in α-MEM (Gibco, United States) supplemented with 10% FBS (Gibco, United States) and 1% penicillin/streptomycin (Gibco, United States).

### 2.4 Lentivirus transduction of DPSCs

Lentiviral vectors for PDGF-BB and GFP were constructed by Cyagen Biosciences, Inc. The target plasmids, LV-EF1a > hPDGFB-CMV > eGFP/T2A/Puro and LV-CMV > eGFP/T2A/Puro (control), were engineered using Gateway technology. For viral packaging, the target plasmid was co-transfected with packaging plasmids (pMDLg/pRRE, pRSV-Rev, and pMD2. G) into 293T cells using Lipofectamine 2000 (Invitrogen, Cat#11668027), following the manufacturer’s protocol. After 48 h, the supernatant was collected and filtered to remove cellular debris. Subsequently, viral particles were concentrated and the viral titer was quantitatively determined. DPSCs at passage 2 were then infected with the lentivirus at a multiplicity of infection (MOI) of 20. Following 72 h of infection, the transduction process was terminated by replacing the medium with fresh growth medium. Successful transduction was confirmed through fluorescence microscopy (Nikon, Japan) by visualizing GFP expression. Based on the transduction conditions, the experimental groups were designated as follows: Control (untransduced cells), GFP (cells transduced with LV-CMV > eGFP/T2A/Puro), and PDGF-BB (cells transduced with LV-EF1a > hPDGFB-CMV > eGFP/T2A/Puro).

### 2.5 Quantitative real-time polymerase chain reaction (qRT-PCR)

Total RNA was extracted using the RNA-Quick Purification Kit (Esunbio, China), followed by reverse transcription into complementary DNA (cDNA) using the PrimeScript™ RT Reagent Kit (Takara, Japan). To quantify the mRNA expression levels of PDGF-BB and VEGF, qRT-PCR was conducted on the Light Cycler 480 II platform (Roche, Germany), with SYBR^®^ Premix Ex Taq™II (Takara, Japan) as the detection reagent. β-Actin was employed as the internal reference gene for mRNA normalization. The relative gene expression levels were determined using the comparative threshold cycle (Cq) method (2^−ΔΔCt^). All primer sequences utilized in this investigation are systematically listed in Table 1.

**TABLE 1:** Primers for mRNA real-time polymerase chain reaction.

Gene	Accession number	Primer (5ʹ to 3ʹ)
***PDGFB***	NM_002608.4	Forward:CTGCGACCTG TCCAGGTGAG
		Reverse:GCACCGTCCGAATGGTCACC
***VEGFA***	NM_001025366.3	Forward:AGGGCAGAATCATCACGAAGT
		Reverse:AGGGTCTCGATTGGATGGCA
***β-Actin***	NM_001101.5	Forward:TGGCACCCAGCACAATGAA
		Reverse:CTAAGTCATAGTCCGCCTAGAAGCA

### 2.6 Western blotting assay

Total cellular proteins were extracted using RIPA lysis buffer (Beyotime, P0038) supplemented with protease and phosphatase inhibitor cocktail (Epizyme, GRF103) to maintain protein integrity. Protein concentrations were determined using a BCA Protein Assay Kit (Thermo Scientific, 23225) according to the manufacturer’s protocol. Equal amounts of protein lysates (20 μg per lane) were separated by electrophoresis on 4%–20% gradient Precast Protein Plus Gels (Yeasen, 36270ES10) and subsequently transferred onto PVDF membranes (Millipore, United States). For immunoblotting analysis, membranes were blocked with 5% bovine serum albumin (BSA) for 1 h at room temperature, followed by overnight incubation at 4°C with the following primary antibodies: anti-PDGF B (1:800 dilution, Abcam, ab23914), anti-VEGFA (1:1000 dilution, Proteintech, 19003-1-AP), anti-β-Actin (1:20000 dilution, Proteintech, 66009-1-Ig), and anti-GAPDH (1:50000 dilution, Proteintech, 60004-1-Ig). After three washes with TBST buffer (Epizymes, PS103), membranes were incubated with HRP-conjugated secondary antibodies (Beyotime, China) for 1 h at room temperature. Following extensive washing, protein bands were visualized using enhanced chemiluminescence (ECL) substrate and imaged using a gel documentation system (Uvitec, United Kingdom).

### 2.7 Enzyme-linked immunosorbent assay (ELISA)

Cells were seeded in 6-well plates at a density of 1 × 10^6^ cells/well and cultured in complete growth medium. Supernatants and cells were harvested at 24, 48, and 72 h post-seeding. Following supernatant collection, adherent cells were trypsinized and counted to determine cell numbers for normalization. The concentrations of PDGF-BB and VEGFA in conditioned media were measured using human PDGF-BB and VEGFA ELISA kits (Multi Sciences, China) following the manufacturer’s instructions. Supernatants were centrifuged (300×g, 5 min) to remove cellular debris prior to analysis. Absorbance was measured at 450 nm with 570 nm as reference wavelength using a microplate reader. Cytokine concentrations were calculated based on standard curves generated with recombinant proteins. To account for potential variations in cell numbers, all measured concentrations were normalized to 1 × 10^6^ cells by dividing the raw concentration values by the actual cell count for each well.

### 2.8 5-ethynyl-2′-deoxyuridine (EdU) incorporation assay

For cell proliferation analysis, cells were seeded in 24-well culture plates at a density of 2.5 × 10^4^ cells/mL in complete growth medium and allowed to adhere for 24 h under standard culture conditions (37°C, 5% CO_2_). Following the initial incubation period, cells were pulse-labeled with 10 µM EdU (Beyotime, C0078S) for 6 h to incorporate the thymidine analog into newly synthesized DNA. After EdU labeling, cells were fixed with 4% paraformaldehyde and subjected to click reaction solution followed by staining with DAPI (Beyotime,C1005). The stained cells were then examined and imaged under a fluorescence microscope. Quantitative analysis of cell proliferation was performed by calculating the percentage of EdU-positive cells relative to the total number of DAPI-stained nuclei. Five randomly selected fields of view (×200 magnification) were analyzed for each experimental condition, and the proliferation rate was expressed as the ratio of EdU-positive nuclei (red fluorescence) to total nuclei (blue fluorescence). All quantitative analyses were performed using ImageJ software (NIH, United States) with appropriate threshold settings to ensure accurate cell counting.

### 2.9 Cell counting Kit-8 assay

Cells were seeded in a 96-well plate at 2000 cells/well density and cultured in growth medium. Cell proliferation curve was assessed at 24-h intervals on days 1, 3, 5, 7, and 9. Prior to each measurement, culture medium was carefully aspirated and the cells were washed with PBS. The CCK-8 assay kit (Dojindo, CK04) was used according to the manufacturer’s instructions to prepare the working solution (1:10 with serum-free DMEM). 100μL of the working solution was added to each well, followed by incubation at 37°C for 1 h. Optical density (OD) values were measured at 450 nm using a microplate reader (TECAN Spark, Switzerland). Relative absorbance values were then employed for normalization, and proliferation curves were subsequently plotted.

### 2.10 Cell cycle assay

Cell cycle distribution of cells was detected by Cell Cycle Analysis Kit (Beyotime, C1052). Cells were resuspended as single cell suspensions and fixed with 70% cold ethanol overnight. After washing with pre-cold PBS, the cells were incubated with propidium iodide (PI)/RNase A at 37°C in the dark for 30 min. Subsequently, red fluorescence was detected at an excitation wavelength of 488 nm using a flow cytometer (Beckman,United States). The statistical data was analyzed using FlowJo software (BD Biosciences, United States).

### 2.11 Transwell migration assay

To evaluate the impact of PDGF-BB overexpression on the migratory capacity of DPSCs, a transwell migration assay was conducted. Briefly, 2.5 × 10^4^ cells/well from three experimental groups (Control, GFP, and PDGF-BB) were seeded into the upper chamber of transwell inserts (8 μm pore size; Corning, 3422) containing serum-free DMEM medium. The lower chamber was filled with DMEM medium supplemented with 5% FBS as a chemoattractant. Following incubation at 37°C with 5% CO_2_ for 12 and 24 h, the migrated cells were fixed with 4% paraformaldehyde for 30 min and subsequently stained with 1% crystal violet solution (Beyotime, C0121) for 15 min. Non-migratory cells remaining in the upper chamber were carefully removed using a cotton swab. After PBS washes, the migrated cells on the lower surface of the membrane were quantified through microscopic imaging and analyzed using ImageJ software.

To further investigate the endothelial cell recruitment potential of the three experimental groups, 5 × 10^4^ cells/well from each group (Control, GFP, and PDGF-BB) were plated in the lower chamber of a transwell system. Following cell adhesion and growth, HUVECs were seeded into the upper chamber at a density of 2.5 × 10^4^ cells/well. After 12 and 24 h of co-culture, the migrated HUVECs were processed using identical fixation, staining, and imaging protocols as described above.

### 2.12 Tube formation assay

To assess the pro-angiogenic potential of the three experimental groups (Control, GFP and PDGF-BB) on HUVECs, an *in vitro* tube formation assay was conducted. The experimental procedure was performed as follows: cells from each group were cultured in standard conditions until reaching 80%–90% confluence. Following PBS washes, the cells were maintained in serum-free DMEM supplemented with 1% penicillin-streptomycin for 48 h to generate conditioned media. The collected supernatant underwent sequential centrifugation at 3000 g for 5 min and 1,500 g for 5 min at 4°C, followed by filtration through a 0.22-μm membrane to obtain cell-free conditioned media, which were stored at 4°C for subsequent experiments.

For the tube formation assay, HUVECs were plated at a density of 5 × 10^4^ cells per well in 96-well plates pre-coated with 60 μL of growth factor-reduced Matrigel (BD Biosciences, 354234). Each well was supplemented with 100 μL of the corresponding conditioned medium from the three experimental groups. Following 6 h of incubation at 37°C with 5% CO_2_, five random fields per well were imaged using an inverted phase-contrast microscope. Quantitative analysis of tube formation was performed using ImageJ software, with particular attention to network parameters such as total tube length and branch points.

### 2.13 Immunofluorescence staining

The expression of relevant proteins after transfection with the PDGF-BB gene was observed using cellular immunofluorescence. The three experimental groups (Control, GFP and PDGF-BB) were cultured on confocal dishes and fixed with 4% paraformaldehyde, followed by permeabilization with 0.1% Triton-100 for 3 min. Non-specific binding sites were blocked using immunostaining blocking solution (Beyotime, P0260) for 15 min at room temperature. Primary antibodies anti-PDGF-B (1:200 dilution; Abcam, ab23914) and VEGFA (1:200 dilution; Proteintech, 19003-1-AP) were incubated overnight at 4°C. On the following day, cells were incubated with Alexa Fluor 594-conjugated secondary antibodies (Yeasen, 34212ES60). The nuclei and cytoskeleton were counterstained with DAPI and phalloidin (Yeasen, 40735ES75), respectively. Images were captured using a laser confocal scanning microscope (Leica, Germany).

### 2.14 Cell seeding and *in vivo* transplantation

To evaluate the *in vivo* angiogenic potential of PDGF-BB gene-modified DPSCs, a kidney capsule transplantation model was established using Sprague-Dawley (SD) rats. GFP or PDGF-BB cells were seeded onto porous calcium phosphate cement (CPC; Rebone Biomaterials, China) scaffolds. Following 24 h of culture, cell adhesion and proliferation on the scaffolds were assessed using confocal microscopy.

All animal experiments were conducted in accordance with the guidelines approved by the Institutional Animal Care and Use Committee of the Ninth People’s Hospital, Shanghai, China. Male SD rats (8–10 weeks old) were obtained from the Animal Center of the Ninth People’s Hospital. The experimental design included three groups: (1) CPC alone (control), (2) CPC with GFP, and (3) CPC with PDGF-BB (n = 4 per group). The cell-scaffold constructs were surgically implanted beneath the left kidney capsule of each rat under sterile conditions. Postoperative care and monitoring were performed according to standard protocols to ensure animal welfare and experimental validity.

### 2.15 Histological analysis

Tissue samples were harvested 8 weeks post-implantation for histological and immunofluorescence analyses. The collected specimens were immediately fixed in 4% paraformaldehyde for 24 h, followed by decalcification in 10% EDTA solution (pH = 7.4) for 7 days with daily solution changes. Subsequently, the samples underwent sequential processing through graded ethanol series (70%, 80%, 90%, and 100%) for dehydration, xylene for clarification, and paraffin embedding. Serial sections of 4 μm thickness were prepared using a microtome for subsequent staining procedures.

For histological evaluation, tissue sections were stained with hematoxylin and eosin (H&E) according to standard protocols. The stained sections were examined under light microscopy to assess tissue regeneration and structural organization. Masson’s trichrome staining was performed using a commercial kit (Servicebio, China) according to the manufacturer’s instructions. Briefly, nuclei were stained with Weigert’s iron hematoxylin for 10 min at room temperature, followed by differentiation in 1% acid alcohol for 30 s and bluing in running tap water for 10 min. Subsequently, sections were stained with Biebrich scarlet-acid fuchsin for 5 min, treated with phosphomolybdic-phosphotungstic acid solution for 10 min, and counterstained with aniline blue for 5 min. Finally, sections were briefly rinsed in 1% acetic acid for 30 s, dehydrated through graded ethanol, cleared in xylene, and mounted with neutral balsam. Stained sections were imaged using a microscope under consistent brightfield illumination (×20objective). Collagen deposition (blue staining) was quantified using ImageJ software with the color deconvolution plugin. To evaluate angiogenesis, immunofluorescence staining was performed on parallel sections. Tissue sections were deparaffinized in xylene and rehydrated through a descending ethanol series. Antigen retrieval was achieved through pepsin treatment. After blocking, the sections were incubated with primary antibody against CD31 (1:2000 dilution; abcam, ab281583) overnight at 4°C. Following PBS washes, sections were incubated with Alexa Fluor 594-conjugated secondary antibody for 1 h at room temperature in the dark. Nuclei were counterstained with DAPI for 5 min. Images were captured using a confocal microscope.

### 2.16 Statistical analysis

All data are presented as the mean ± standard deviation (mean ± SD) of at least three independent repeated experiments. When a normal data distribution was confirmed, data were analyzed by Student’s t-test and one way analysis of variance (ANOVA) in GraphPad Prism 9.0. (La Jolla, CA, United States). Data with a p value of <0.05 were considered statistically significant. All the experiments were independently repeated at least three times.

## 3 Result

### 3.1 PDGF (+) mesenchymal stem cells were identified by scRNA-seq and exhibits stronger intercellular communication with endothelial cells

**FIGURE 1:**
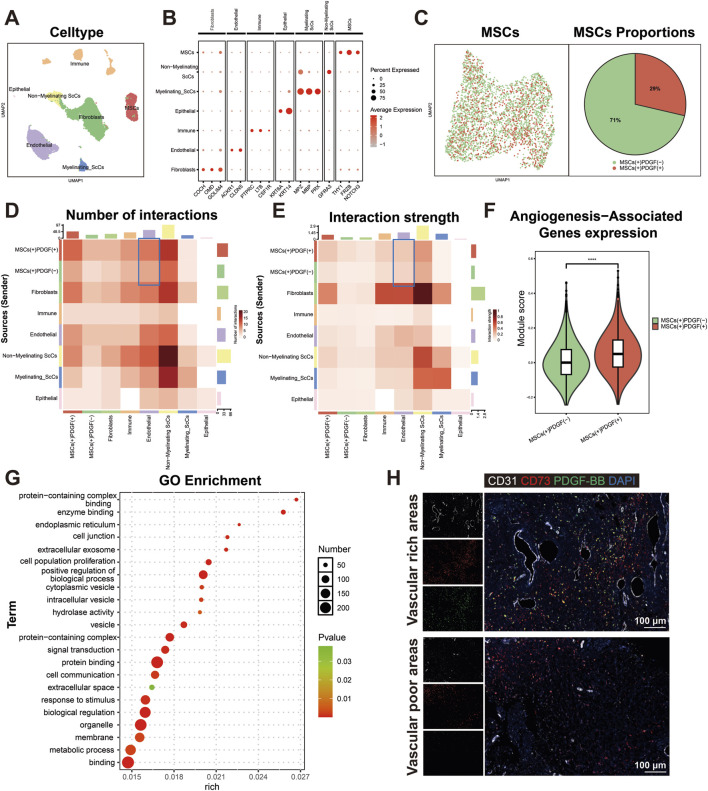
Identification and characterization of PDGF(+) MSCs in dental pulp angiogenesis. **(A)** UMAP visualization of seven distinct cell populations identified through single-cell RNA sequencing analysis. **(B)** Dotplot representation of characteristic marker genes used for cell type annotation and cluster identification. **(C)** Proportional distribution of PDGF (+) and PDGF (−) subpopulations within the MSC population, presented as a percentage pie chart. **(D, E)** Cell interaction analysis of each cell in dental pulp tissue. MSC (+) PDGF (+) cells exhibited significantly stronger intercellular interaction with endothelial cells compared to MSC (+) PDGF (−) cells.**(F)** Comparative analysis of angiogenesis-associated gene expression patterns between PDGF (+) and PDGF (−) subpopulations, presented as module scores (****p < 0.0001). **(G)** The results of GO enrichment analysis of DEGs upregulated in PDGF (+) cells compared to PDGF (−) cells, highlighting significant biological processes. **(H)** mIHC staining of human dental pulp tissue showing spatial distribution of CD31 (white; endothelial cells), CD73 (red; mesenchymal stem cells), PDGF-BB (green), and DAPI (blue; nuclei). Scale bar = 100 μm.

To elucidate the specific subpopulation of DPSCs involved in dental pulp angiogenesis, we conducted comprehensive analysis of scRNA sequencing data from dental pulp tissue. After quality control and cell filtering, cells were classified into seven distinct clusters based on gene expression profiles (Figures 1A, B). Utilizing the Seurat clustering workflow, MSCs were further subdivided into two subpopulations according to PDGF gene expression patterns. Quantitative analysis revealed that PDGF-negative (PDGF (−)) cells constituted 71% of the total MSC population, while PDGF-positive (PDGF (+)) cells accounted for the remaining 29% (Figure 1C). To investigate potential cellular interactions, we employed Cellchat analysis to analyze communication networks between these MSC subpopulations and endothelial cells. The analysis demonstrated that PDGF (+) cells exhibited significantly enhanced intercellular communication compared to PDGF (−) cells, as evidenced by both the number of interactions (Figure 1D) and interaction strength (Figure 1E). This observation suggests that PDGF (+) MSCs may play a more active role in cellular crosstalk with endothelial cells. Furthermore, angiogenesis-associated gene expression profiling revealed significantly higher module scores in PDGF (+) cells compared to PDGF (−) cells (*p* < 0.0001) (Figure 1F), GO enrichment analysis of DEGs between the two subpopulations demonstrated that upregulated DEGs in PDGF (+) cells were significantly enriched in biological processes related to cell population proliferation, extracellular exosome formation, signal transduction, cell communication, and response to external stimuli (Figure 1G). These findings suggest that PDGF (+) cells may play a crucial role in intercellular signaling pathways and participate in tissue repair mechanisms following injury. To further characterize the spatial distribution of PDGF (+) MSCs within dental pulp tissue, we performed mIHC staining on healthy human dental pulp samples (Figure 1H). CD73 was utilized as a mesenchymal stem cell marker, while CD31 served as an endothelial cell marker. Our result revealed a distinct niche localization pattern, with CD73 (+) PDGF (+) cells predominantly localized in perivascular regions of vascular-rich areas. In contrast, vascular-poor regions were exclusively populated by CD73 (+) PDGF (−) cells. The proportion of PDGF (+) cells within a 15 μm radius of the nearest blood vessel was approximately twice that of PDGF (−) cells (see Supplementary Figure S1 for details). This spatial distribution pattern suggests that the PDGF (+) MSC subpopulation may be specifically associated with vascular structures, potentially contributing to angiogenesis or vascular stabilization processes in dental pulp tissue.

### 3.2 Efficient *PDGFB* gene transfection was carried out using lentiviral vectors after the identification of DPSCs

The identity of isolated DPSCs was initially confirmed through flow cytometry analysis (Supplementary Figure S2A). The results showed that the obtained cells exhibited high expression of MSC surface markers: CD73 (99.4%), CD105 (99.1%), and CD90 (98.4%). Conversely, hematopoietic lineage markers CD14, CD45, and CD34 showed minimal expression, with positive rates of only 0.041%, 2.01%, and 0.057%, respectively. After 7 and 21 days of osteogenic induction, alkaline phosphatase staining revealed distinct blue coloration (Supplementary Figure S2B), while Alizarin Red S staining demonstrated extensive formation of orange-red calcium nodules (Supplementary Figure S2C) in contrast to the non-induced control group which showed minimal staining. After 28 days of adipogenic induction, oil Red O staining confirmed the presence of numerous orange-red lipid droplets, indicating successful adipogenic differentiation (Supplementary Figure S2D). These results collectively demonstrate the osteogenic and adipogenic differentiation potential of the isolated DPSCs.

**FIGURE 2:**
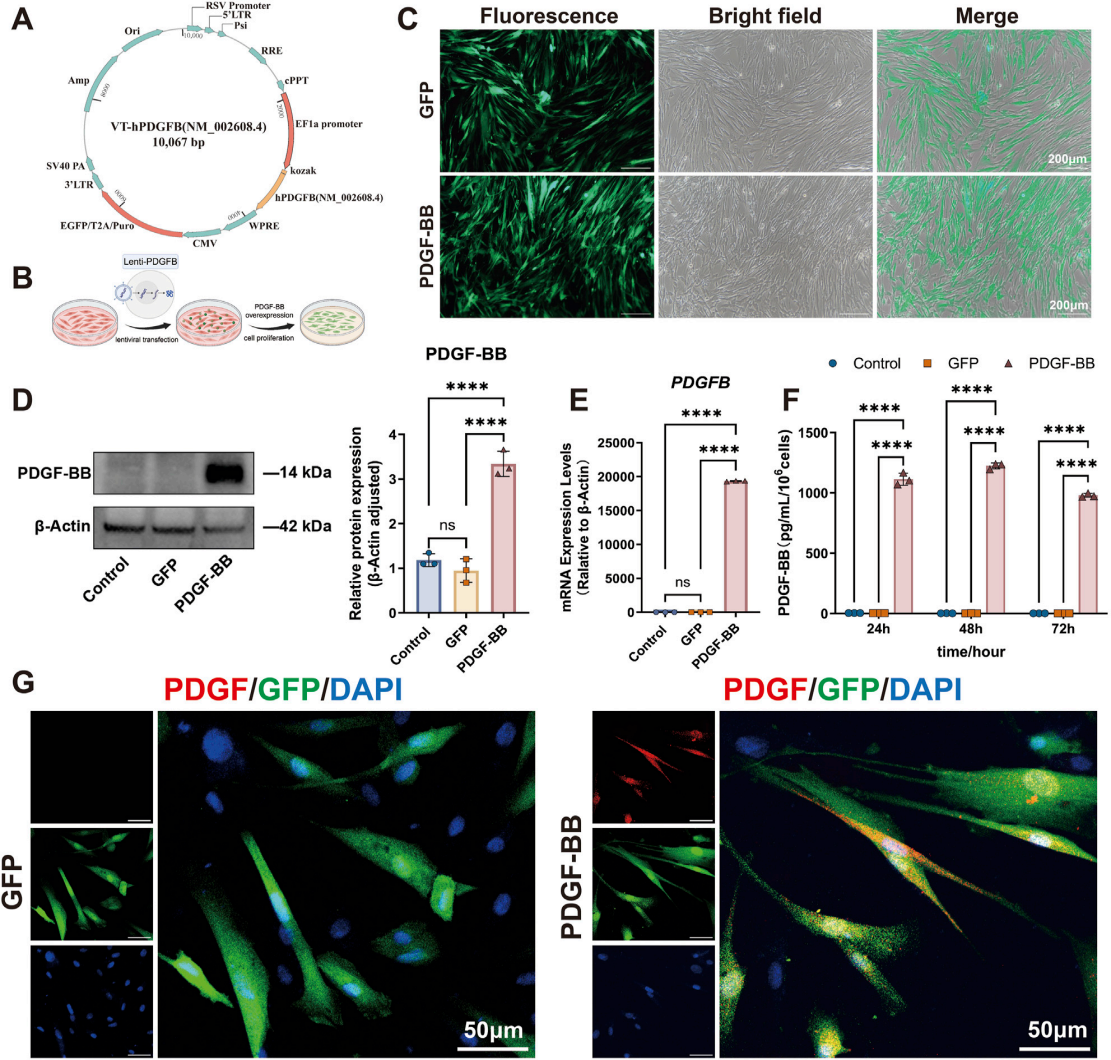
Lentiviral transfection significantly enhanced both the gene and protein expression levels of PDGF-BB in DPSCs. **(A, B)** Schematic diagram of the lentiviral plasmid for overexpression of PDGF-BB and lentiviral transfection process. **(C)** Observation under Fluorescence microscope after transfection of 72 h. Scale bar = 200 μm. **(D)** Western blot analysis were used to measure PDGF-BB protein level. (n = 3) **(E)** qRT-PCR analysis were used to measure PDGF-BB mRNA level. (n = 3) **(F)** ELISA analysis were used to measure PDGF-BB secretion level. (n = 3) **(G)** Immunofluorescence detection of PDGF-BB expression. Scale bar = 50 μm. (ns, no significant difference; ****p < 0.0001).

Figure 2A illustrates the schematic structure of the lentiviral overexpression plasmid for PDGF-BB, while Figure 2B outlines the key steps of the lentiviral transfection process. Transfection efficiency was assessed 72 h post-transduction by fluorescence microscopy. Both the empty vector control group (GFP) and PDGF-BB overexpression group (PDGF-BB) demonstrated approximately 80% transfection efficiency, as evidenced by green fluorescent protein expression (Figure 2C). To confirm successful PDGF-BB overexpression, total cellular proteins and RNA were extracted for subsequent analysis. Western blot analysis revealed a significant three-fold increase in PDGF-BB protein expression in the PDGF-BB group compared to both Control and GFP groups (*p* < 0.0001, Figure 2D). Consistent with these findings, qRT-PCR analysis demonstrated a remarkable 19,000-fold upregulation of PDGF-BB mRNA expression levels in the PDGF-BB group relative to controls (*p* < 0.0001, Figure 2E). The functional consequence of this genetic modification was confirmed through ELISA quantification of secreted PDGF-BB. Transfected DPSCs exhibited sustained PDGF-BB secretion, with extracellular protein levels reaching 1000-fold higher concentrations than both control groups (*p* < 0.0001, Figure 2F). Immunofluorescence staining provided additional confirmation, revealing that while GFP transfection did not alter endogenous PDGF-BB expression levels, PDGF-BB-transfected cells displayed substantially enhanced red fluorescence intensity, indicating successful overexpression of the target protein (Figure 2G).

### 3.3 PDGF-BB overexpressing DPSCs has improved proliferation and migration ability

**FIGURE 3:**
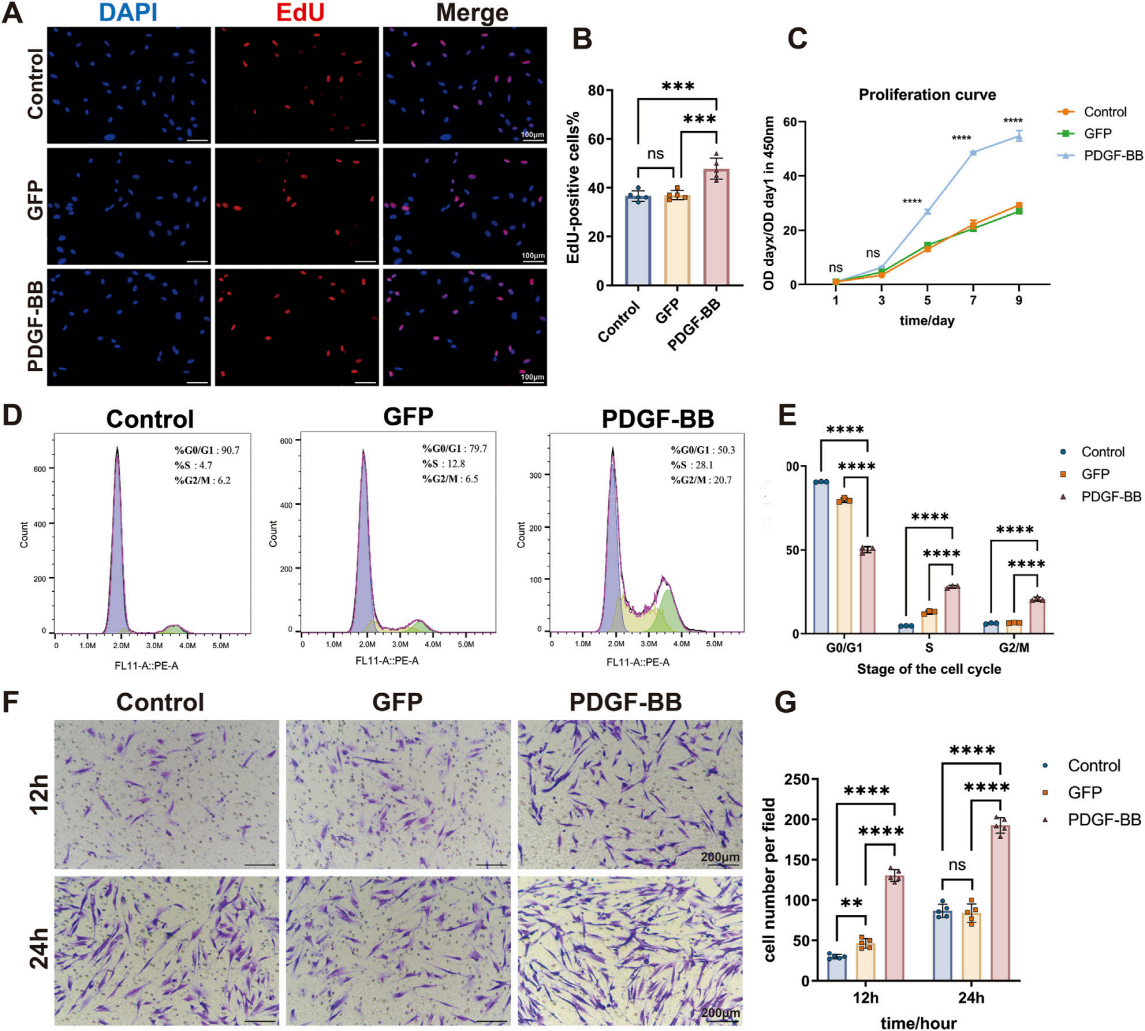
PDGF-BB overexpressing enhanced the proliferation and migration ability of DPSCs. **(A, B)** Representative images and quantitative analysis of EdU incorporation assay. Cells in the process of DNA replication are stained red, and the percentage of EdU-positive cells was calculated as the ratio of EdU-positive cells to the total number of nuclei stained with DAPI (blue). Scale bar = 100 μm (n = 5). **(C)** Cell proliferation curves were analyzed using the CCK-8 assay. (n = 3) **(D)** Cell cycle distribution analyzed by flow cytometry. **(E)** Quantitative analysis of the cell cycle distribution. The percentages of cells in G0/G1, S, and G2/M phases for each group are presented as bar graphs. (n = 3) **(F, G)** Representative images and quantitative analysis of the transwell migration assay. Scale bar = 200 μm. (n = 5) (ns, no significant difference; *p < 0.05; **p < 0.01; ***p < 0.001; ****p < 0.0001).

To explore the effect of PDGF-BB overexpressing on the proliferation ability of DPSCs, we conducted a comprehensive analysis using EdU incorporation and CCK-8 assays to compare cellular proliferation across three experimental groups. Quantitative analysis demonstrated that during the logarithmic growth phase, the PDGF-BB group displayed a significantly increased number of EdU-positive cells compared to both the Control and GFP groups (*p* < 0.0001, Figures 3A, B), while no significant difference was observed between the Control and GFP groups, indicating that lentiviral transfection *per se* does not affect cellular proliferation. These findings were corroborated by the proliferation curve analysis, which revealed a substantial elevation in the PDGF-BB group compared to control groups, with the proliferation rate in the PDGF-BB group reaching twice that of the other groups after day 5 (*p* < 0.0001, Figure 3C).

Cell cycle analysis provided additional mechanistic insights, revealing that PDGF-BB overexpression induced significant changes in cell cycle distribution. The PDGF-BB group exhibited a marked decrease in the proportion of cells in the G0/G1 phase, with a corresponding increase in the S and G2/M phase populations (*p* < 0.0001, Figures 3D, E). These data suggest that PDGF-BB overexpression promotes cell cycle progression from G1 to S and G2/M phases, thereby enhancing cellular replication and proliferative capacity. These results are consistent with the findings from the EdU and CCK-8 proliferation assays.

To further elucidate the functional consequences of PDGF-BB overexpression, we assessed cellular migration capacity using transwell assay. Quantitative analysis at 12 and 24-h intervals revealed significantly enhanced migratory activity in the PDGF-BB group. At 12 h, the mean number of PDGF-BB group cells traversing the membrane was 4.3-fold and 2.8-fold higher than that of the Control and GFP groups, respectively. At 24 h, the PDGF-BB group showed approximately twice the number of migrating cells compared to both control groups (*p* < 0.0001, Figures 3F, G). These collective findings demonstrate that PDGF-BB genetic modification not only enhances proliferative potential but also significantly increases the migratory capacity of DPSCs.

### 3.4 PDGF-BB overexpressing enhances the capability of DPSC-mediated angiogenesis and upregulated pro-angiogenesis cytokine VEGFA expression of DPSCs

**FIGURE 4:**
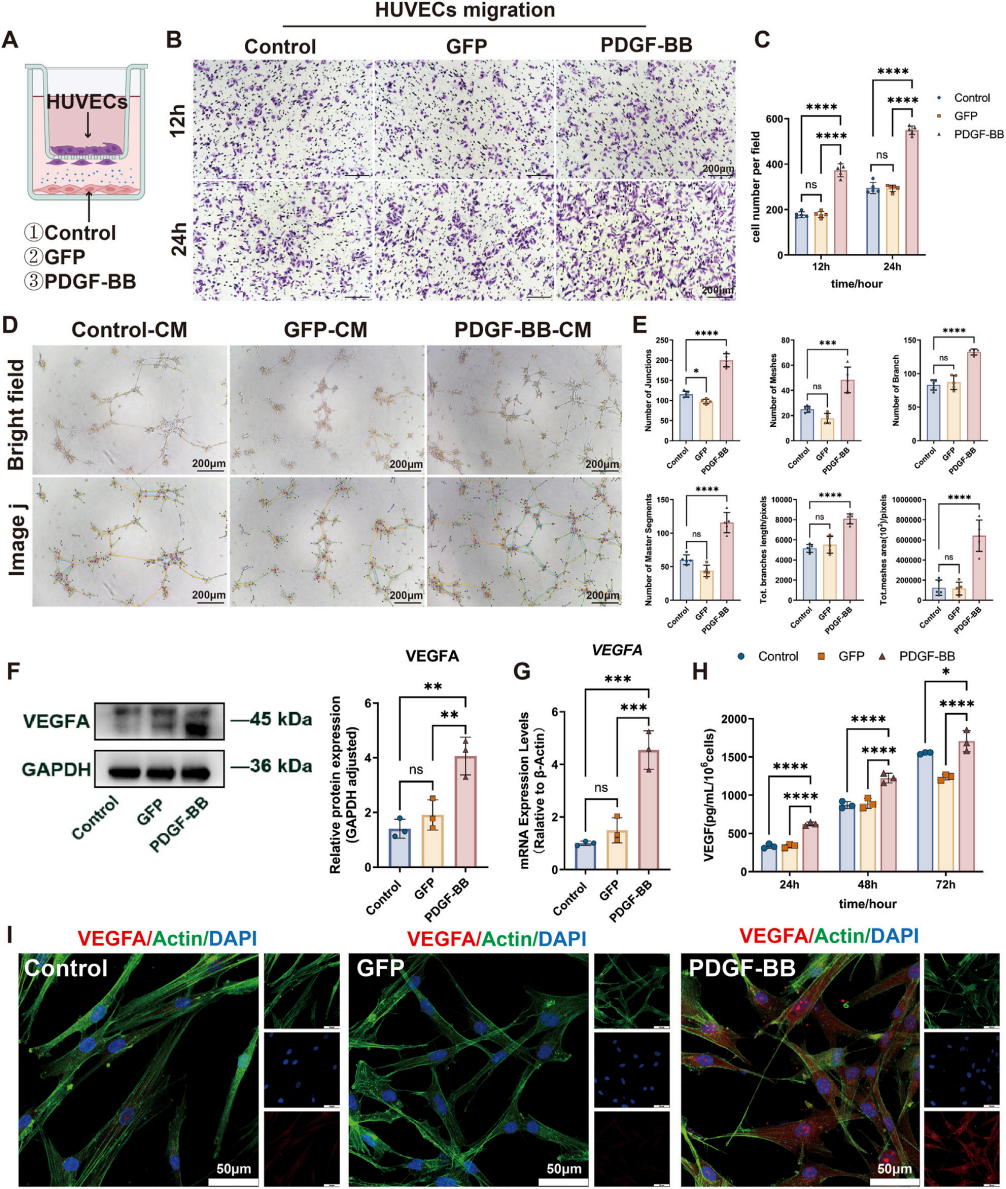
PDGF-BB overexpressing enhances DPSC-mediated angiogenesis by upregulating VEGFA expression. **(A)** Schematic diagram of Control, GFP and PDGF-BB cells coculture with HUVECs for *in vitro* experiments. **(B, C)** Transwell co-culture system was used to evaluate the recruitment ability of DPSC, DPSC-GFP, and DPSC-PDGF on HUVECs migration. Scale bar = 200 μm. (n = 5) **(D, E)** Tube formation assay of HUVECs stimulated with conditioned media from the three experimental groups. Representative images and quantitative analysis of tube-like structures are shown. Scale bar = 200 μm. (n = 5) **(F, G)** mRNA and protein expression levels of VEGFA in different groups. (n = 3) **(H)** ELISA analysis were used to measure VEGFA secretion level. (n = 3) **(I)** Confocal microscope images demonstrate enhanced VEGFA signals in PDGF-BB compared to Control and GFP groups. Scale bar = 50 µm. (ns, no significant difference; *p < 0.05; **p < 0.01; ***p < 0.001; ****p < 0.0001).

The recruitment of vascular endothelial cells and their subsequent formation of tube-like structures constitute the fundamental cytological basis of angiogenesis. To further investigate the regulatory role of PDGF-BB in DPSC-mediated angiogenesis, we established a transwell co-culture system comprising HUVECs and DPSCs to assess whether PDGF-BB overexpression enhances endothelial cell recruitment (Figure 4A). In this system, Control, GFP and PDGF-BB DPSCs were cultured in the lower chamber of the transwell, allowing secreted functional factors to influence HUVECs in the upper chamber through the semi-permeable membrane. After 12 and 24 h of co-culture, the migratory capacity of HUVECs was assessed. Compared to the Control and GFP groups, co-culture with PDGF-BB overexpressing DPSCs significantly enhanced HUVECs migration by approximately twofold (*p* < 0.0001, Figures 4B, C).

In addition, we also collected the supernatants of the three experimental groups and prepared them into conditioned media to stimulate HUVECs. The tube-forming ability of HUVECs was detected on Matrigel. As shown in Figures 4D, E, our results indicated that the overexpression of PDGF-BB, rather than the control vector, significantly promoted the formation of tube-like structures in HUVECs. The number of junctions, number of branches, number of meshes, number of master segments, total meshes area, and total branches length of the tube-like structures all increased significantly. These findings collectively demonstrate that PDGF-BB-overexpressing DPSCs exhibit an enhanced capacity to promote endothelial cell angiogenesis *in vitro*.

Given the pivotal role of VEGFA as a critical angiogenic growth factor in tissue repair and regeneration, we further investigated the impact of PDGF-BB overexpression on VEGFA expression in DPSCs. At the transcriptional level, qRT-PCR analysis demonstrated a significant 4.5-fold increase in VEGFA mRNA expression in PDGF-BB-overexpressing DPSCs compared to both untransfected controls and GFP-transfected cells (*p* < 0.001) (Figure 4G). This transcriptional activation was paralleled at the protein level, with Western blot analysis showing a 2.6-fold elevation in intracellular VEGFA protein (*p* < 0.01) (Figure 4F). To functionally validate the observed VEGFA upregulation, we performed temporal secretion profiling using ELISA. Quantitative analysis demonstrated a time-dependent increase in VEGFA secretion from PDGF-BB-modified DPSCs, with significantly elevated levels (*p* < 0.0001) observed at all measured timepoints (24h, 48h, and 72 h) compared to control groups (Figure 4H). This progressive secretion pattern suggests PDGF-BB overexpression induces durable alterations in the DPSC secretory profile, potentially enhancing their paracrine angiogenic capacity. Consistent with these results, confocal microscopy revealed stronger VEGFA signals in PDGF-BB-overexpressing DPSCs (Figure 4I). These data underscore the mechanistic link between PDGF-BB overexpression and enhanced angiogenic potential via VEGFA upregulation in DPSCs.

### 3.5 *In vivo* transplantation of PDGF-BB overexpressing DPSCs demonstrated enhanced angiogenic potential

**FIGURE 5:**
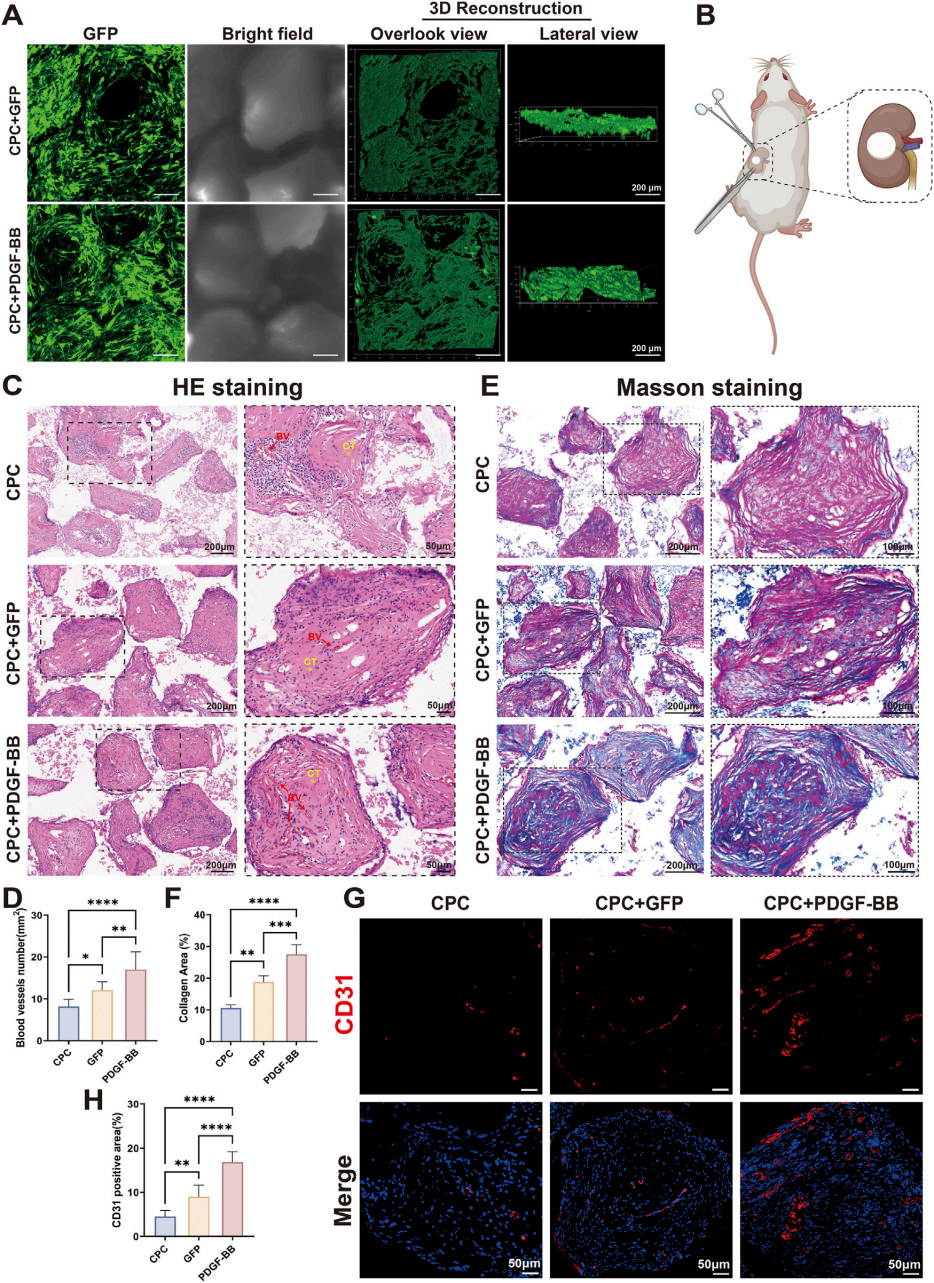
In vivo transplantation of PDGF-BB overexpressing DPSCs demonstrated enhanced angiogenic potential. **(A)** Fluorescence microscopy images of cells seeding on CPC scaffold after culture 24 h in vitro. Scale bar = 100 μm. **(B)** Schematic diagram of the surgery for sub kidney capsule transplantation of tissue-engineered composites. **(C,D)** HE staining of tissue sections and statistical analyses of blood vessel numbers. Scale bar = 200 μm, 50 μm (partial enlargement). (BV, blood vessel; CT, collagenous tissue; n = 10) (E,F) Masson staining of tissue sections and statistical analyses of the percentage of collagen-positive area. Scale bar = 200 μm, 100 μm (partial enlargement). (G,H) Immunofluorescence staining for the angiogenesis marker CD31 expression in groups and statistical analyses of CD31 positive area. Scale bar = 50 μm. (ns, no significant difference; *p < 0.05; **p < 0.01; ***p < 0.001; ****p < 0.0001).

While our *in vitro* studies confirmed the biological functionality of PDGF-BB overexpression, further *in vivo* investigations were essential to evaluate its clinical applicability. Given the superior biocompatibility and cell adhesion properties of CPC materials, we fabricated tissue-engineered composite scaffolds by seeding DPSCs onto CPC substrates. Cellular growth and adhesion were assessed 24 h post-seeding via confocal microscopy (Figure 5A). Three-dimensional reconstruction revealed uniform distribution of DPSCs across the material surface and within porous structures, with established cellular junctions, thereby confirming cell viability for subsequent *in vivo* applications. To evaluate tissue regeneration capacity, we implanted these tissue-engineered composite scaffolds beneath the kidney capsule of rat models (Figure 5B). Histological evaluation via HE staining revealed significantly enhanced vascularization and tissue integration in the PDGF-BB overexpressing group compared to controls (Figures 5C, D). Vascularization in the CPC-only scaffold group was limited, with a microvessel density of 8.2 ± 1.7 vessels/mm^2^. The GFP/CPC composite scaffold group showed a 31.7% increase in vascular density (12.1 ± 2.0 vessels/mm^2^). Notably, the PDGF-BB overexpression group demonstrated superior regenerative performance, with microvessel density further elevated to 17.0 ± 4.2 vessels/mm^2^, representing a 40.5% enhancement over the GFP group (*p* < 0.01). The presence of erythrocytes within the luminal structures of newly formed vessels confirmed functional vascularization, indicative of perfusable blood vessel formation. Masson’s trichrome staining revealed disorganized collagen fibers in the CPC-only group (10.6% ± 1.1% area). The GFP/CPC group exhibited a 77.4% increase in collagen deposition (18.8% ± 1.9%; *p* < 0.01 vs CPC), though fibers remained short and randomly oriented. In contrast, the PDGF-BB overexpression group achieved 27.5% ± 3.0% collagen area (46.0% improvement vs GFP; *p* < 0.001), with fibers adopting a parallel, lamellar arrangement resembling native dental pulp tissue, indicating advanced ECM maturation (Figures 5E, F). Immunofluorescence staining further substantiated these observations, revealing significantly elevated expression of the endothelial marker CD31 in the PDGF-BB group (Figures 5G, H). Collectively, these findings underscore the robust *in vivo* angiogenic capacity of PDGF-BB-overexpressing DPSCs, highlighting their therapeutic potential for vascularized tissue regeneration applications.

## 4 Discussion

DPSCs are pivotal in maintaining dental pulp homeostasis and play a critical role in pulp development and injury repair. Early studies have identified DPSCs within the “stem cell niche” adjacent to blood vessels and nerves, suggesting a regulatory relationship between DPSCs and vascular components (Kok et al., 2022). Investigating the signaling mechanisms governing DPSCs-vascular interactions is therefore essential for addressing vascularization challenges in pulp regeneration. The PDGF/PDGFR signaling pathway is a key regulator of vascular remodeling and homeostasis, with well-documented roles in tumorigenesis and cardiovascular diseases (Cn et al., 2024). Recent work by the Chai group demonstrated that PDGF derived from blood vessels can modulate specific MSC subsets to promote tissue homeostasis and regeneration (Guo et al., 2024). In regenerative endodontic procedures, the introduction of autologous blood into the root canal to form a blood clot scaffold delivers a rich source of growth factors, including PDGF (Yan et al., 2023; Wei et al., 2022). However, the role of PDGF signaling in pulp vascularization during tissue engineering applications remains underexplored. To address this, we analyzed single cell sequencing data from DPSCs and identified two distinct subsets based on PDGF expression. Notably, only 29% of DPSCs expressed PDGF, yet these PDGF (+) cells exhibited elevated expression of angiogenesis-related genes and enhanced interactions with endothelial cells. Furthermore, PDGF (+) DPSCs showed increased expression of genes involved in cell communication and signal transduction, suggesting their potential as a specialized subset with superior angiogenic capacity. These findings align with studies by Di et al. (2024b), who identified a PDGFRβ(+) DPSCs subset critical for pulp vascularization using single-cell pseudotime analysis. Together, these results highlight the importance of PDGF/PDGFR signaling in pulp vascular development and suggest a novel therapeutic strategy: expanding PDGF (+) DPSCs *in vitro* to provide a consistent and effective cell source for clinical applications.

PDGF signaling regulates diverse biological processes, including proliferation, migration, and differentiation. Among the PDGF family, PDGF-BB is unique in its ability to bind all three PDGF receptors (PDGFR-αα, αβ, and ββ), triggering distinct downstream signaling cascades and earning its designation as the “universal isoform” (Evrova and Buschmann, 2017). PDGF-BB is also detectable in the secretome of normal DPSCs (Caseiro et al., 2019), further supporting its relevance in dental pulp biology. Building on these findings, we genetically modified DPSCs to overexpress PDGF-BB, achieving a 19,000-fold increase in expression compared to controls. These modified cells exhibited robust proliferation and enhanced migration *in vitro*, critical attributes for tissue engineering applications. Similar results were reported by Stuart et al., who observed increased proliferation in PDGF-BB overexpressing MSCs, though the effect varied with promoter choice (CMV vs. PGK) (Guzman et al., 2021). In contrast, Chen et al. (2015) found that PGK-driven PDGF-BB expression in hematopoietic stem cells (HSCs) did not enhance proliferation, potentially due to cell type-specific epigenetic modifications (Nieuwenhuis et al., 2021; Norrman et al., 2010). Notably, our PDGF-BB overexpressing DPSCs demonstrated significantly improved migration, consistent with findings by Deng et al. in periodontal ligament stem cells (PDLSCs), where PDGF-BB-mediated migration involved the Wnt/β-catenin pathway (Deng et al., 2021).

To evaluate the angiogenic potential of PDGF-BB overexpressing DPSCs, we established a co-culture system with HUVECs. PDGF-BB overexpressing DPSCs significantly enhanced HUVECs migration and tube formation, demonstrating their ability to promote vascular regeneration *in vitro*. These results are supported by Zhang et al., who showed that PDGF-BB promotes capillary-like structure formation and smooth muscle cell recruitment in co-cultures of DPSCs and human umbilical artery smooth muscle cells (HUASMCs) (Zhang et al., 2022). Our data demonstrates that PDGF-BB gene modification significantly enhances the angiogenic potential of DPSCs, primarily through upregulation of VEGFA expression and secretion. This aligns with prior studies identifying VEGFA as the most abundant and functionally critical angiogenic factor in DPSCs secretomes (Caseiro et al., 2019; Ge et al., 2018). In addition to VEGFA, MSCs secrete hepatocyte growth factor (HGF) and fibroblast growth factor (FGF), which play pivotal roles in promoting angiogenesis through distinct yet complementary mechanisms. FGF-2 is known to synergize with VEGFA in endothelial cell proliferation (Khan et al., 2017), and HGF may promote vascular maturation (Tang et al., 2018; Kobayashi et al., 2006). However, existing literature suggests that VEGFA is the dominant mediator in DPSC-driven angiogenesis due to its high basal expression and potent endothelial-specific effects (Su su et al., 2025; Ge et al., 2018). Notably, our ELISA data confirmed that PDGF-BB-overexpressing DPSCs secrete significantly higher levels of both PDGF-BB and VEGFA, supporting the paracrine mechanism proposed. Complementary qRT-PCR and Western blot analyses further validated the consistency of VEGFA upregulation across transcriptional and translational levels. For *in vivo* validation, we employed a novel ectopic pulp regeneration model using the sub-kidney capsule site. The kidney capsule, a robust connective tissue membrane, provides stable material fixation and creates an isolated microenvironment that minimizes cellular interference from surrounding tissues. Furthermore, the kidney’s extensive vascular network ensures optimal nutrient supply, promoting cell survival and proliferation while facilitating the investigation of cellular interactions and vascularization processes. (Scott et al., 2012; Piglionico et al., 2023). Tissue-engineered composites of DPSCs and CPC were transplanted beneath the kidney capsule of rats. The PDGF-BB overexpressing group exhibited remarkable angiogenesis, increased pulp-like collagen deposition, and reduced immune cell infiltration compared to controls. These observations are consistent with previous studies: Stuart et al. demonstrated enhanced osteogenesis and angiogenesis in PDGF-BB overexpressing MSCs encapsulated in hydrogels (Guzman et al., 2021), and Zhang et al. observed dentin-like mineralization in subcutaneous transplants of PDGF-BB overexpressing DPSCs(M. Zhang et al., 2017). Although significant mineralization was not observed in our study, the enhanced collagen deposition and vascularization suggest that PDGF-BB overexpressing DPSCs represent a promising cell source for pulp regeneration. The potent angiogenic activity of PDGF-BB is mediated through multiple signaling pathways. Upon binding to its receptors (PDGFR-α/β), PDGF-BB primarily activates the PI3K/Akt pathway, which promotes endothelial cell survival and migration by inhibiting apoptosis and enhancing cytoskeletal reorganization (Zheng et al., 2019). PDGF-BB stimulation also produced an acute induction of TGF-beta expression in SMCs through the MAPK/ERK pathway to maintain vascular homeostasis (Nishishita and Lin, 2004). These findings highlight the dual role of PDGF-BB in promoting both angiogenesis and extracellular matrix remodeling.

However, despite the demonstrated ability of genetic modification to enhance the secretion of angiogenesis-related factors by DPSCs, the construction of an efficient and stable vascular network within the root canal remains a significant challenge. Future research should focus on: (1) Developing next-generation gene delivery platforms (e.g., non-integrating episomal vectors or transient mRNA transfection) to achieve controlled PDGF-BB expression while minimizing genomic integration risks; (2) Implementing inducible expression systems to ensure spatiotemporal control of therapeutic factor secretion; (3) Gaining deeper understanding of the *in vivo* behavior of PDGF-BB overexpressing DPSCs through advanced lineage tracing and single-cell RNA sequencing. Elucidating the precise mechanisms by which genetically modified DPSCs interact with host endothelial cells to promote endogenous vascular regeneration. Such insights are essential for ensuring consistent outcomes across diverse patient populations and treatment protocols. Additionally, further studies should assess whether transient genetic modifications can retain therapeutic efficacy while mitigating long-term risks. Large animal models and clinical trials are essential to validate the safety and efficacy of engineered DPSCs for pulp regeneration. These steps will be critical for translating these promising findings into clinically viable therapies.

## 5 Conclusion

In summary, our findings indicate that DPSCs expressing PDGF signaling in dental pulp tissue may be associated with angiogenesis process. Genetic modification and *in vitro* expansion of PDGF-BB overexpressing DPSCs enhanced endothelial cell homing and tube formation, as well as promoted vascularized pulp regeneration *in vivo*. These results provide valuable insights into the mechanisms of PDGF-BB-mediated vascularization and underscore the therapeutic potential of PDGF-BB overexpressing DPSCs for pulp regeneration. Collectively, this study enhances our understanding of the clinical applications of genetically engineered DPSCs and establishes a strong foundation for the development of personalized and efficient pulp regeneration therapies.

## Data Availability

The datasets presented in this study can be found in online repositories. The names of the repository/repositories and accession number(s) can be found below: GSE164157.
