# Correction: PDGF-BB overexpressing dental pulp stem cells improve angiogenesis in dental pulp regeneration

**DOI:** 10.3389/fbioe.2026.1819960

**Published:** 2026-04-02

**Authors:** Wentao Jiang, Shuhan Duan, Weiping Li, Huijiao Yan, Chenli Si, Ningwei Xu, Yishuai Li, Wenjie Zhang, Shensheng Gu

**Affiliations:** 1 Department of Endodontics, Shanghai Ninth People’s Hospital, Shanghai Jiao Tong University School of Medicine, Shanghai, China; 2 College of Stomatology, Shanghai Jiao Tong University, Shanghai, China; 3 National Center for Stomatology and National Clinical Research Center for Oral Diseases, Shanghai, China; 4 Shanghai Key Laboratory of Stomatology and Shanghai Research Institute of Stomatology, Shanghai, China; 5 Department of Oral Surgery, Shanghai Ninth People’s Hospital, Shanghai Jiao Tong University School of Medicine, Shanghai, China; 6 The Affiliated Stomatological Hospital of Nanjing Medical University, State Key Laboratory Cultivation Base of Research, Prevention and Treatment for Oral Diseases, Jiangsu Province Engineering Research Center of Stomatological Translational Medicine, Nanjing, China; 7 Department of Prosthodontics, Shanghai Ninth People’s Hospital, Shanghai Jiao Tong University School of Medicine, Shanghai, China; 8 Department of Dermatology, Shanghai Ninth People’s Hospital, Shanghai Jiao Tong University School of Medicine, Shanghai, China

**Keywords:** dental pulp stem cells, vascularization, single-cell RNA sequencing, endothelial, dental pulp regeneration

There was a mistake in Figure 5A as published. During the final figure assembly, an incorrect representative image was inadvertently inserted into Figure 5A. The corrected Figure 5 appears below.

**FIGURE 5:**
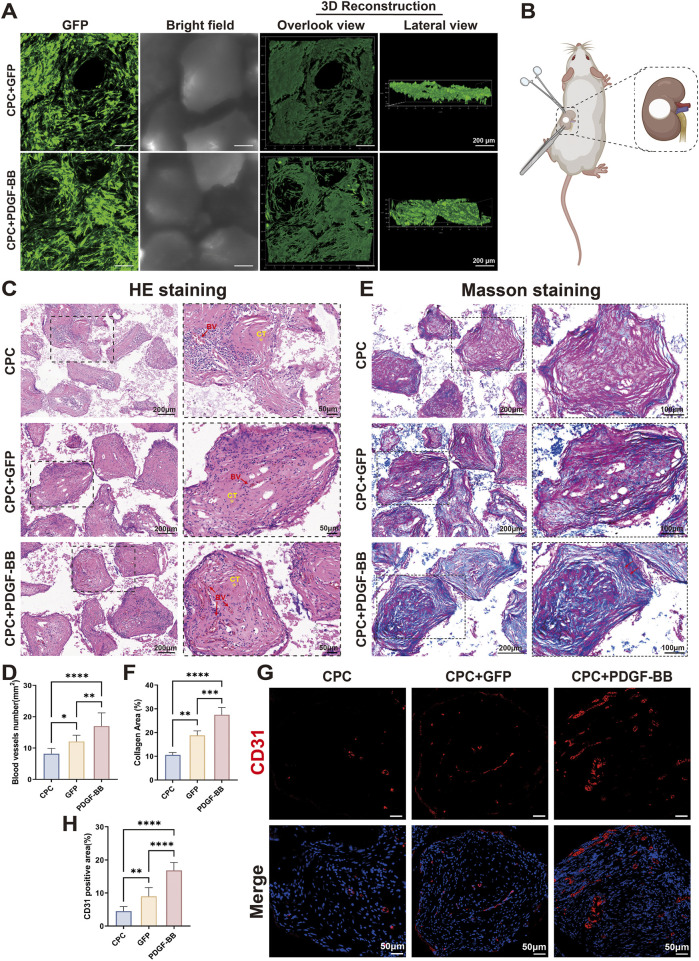
*In vivo* transplantation of PDGF-BB overexpressing DPSCs demonstrated enhanced angiogenic potential. **(A)** Fluorescence microscopy images of cells seeding on CPC scaffold after culture 24 h *in vitro*. Scale bar = 100 μm. **(B)** Schematic diagram of the surgery for sub kidney capsule transplantation of tissue-engineered composites. **(C,D)** HE staining of tissue sections and statistical analyses of blood vessel numbers. Scale bar = 200 μm, 50 μm (partial enlargement). (BV, blood vessel; CT, collagenous tissue; n = 10) **(E,F)** Masson staining of tissue sections and statistical analyses of the percentage of collagen-positive area. Scale bar = 200 μm, 100 μm (partial enlargement). **(G,H)** Immunofluorescence staining for the angiogenesis marker CD31 expression in groups and statistical analyses of CD31 positive area. Scale bar = 50 μm. (ns, no significant difference; *p < 0.05; **p < 0.01; ***p < 0.001; ****p < 0.0001).

The original article has been updated.

## Generated AI statement

Any alternative text (alt text) provided alongside figures in this article has been generated by Frontiers with the support of artificial intelligence and reasonable efforts have been made to ensure accuracy, including review by the authors wherever possible. If you identify any issues, please contact us.

