# Supplementary material for: PDGF-BB overexpressing dental pulp stem cells improve angiogenesis in dental pulp regeneration
Source: Front Bioeng Biotechnol. 2025 Apr 24;13:1578410. doi: 10.3389/fbioe.2025.1578410 (PMC12058851; doi:10.3389/fbioe.2025.1578410)
Supplement: Supplementary file 1 [file DataSheet1.docx]

Supplementary Material

# Supplementary Figures

## Supplementary Figure 1


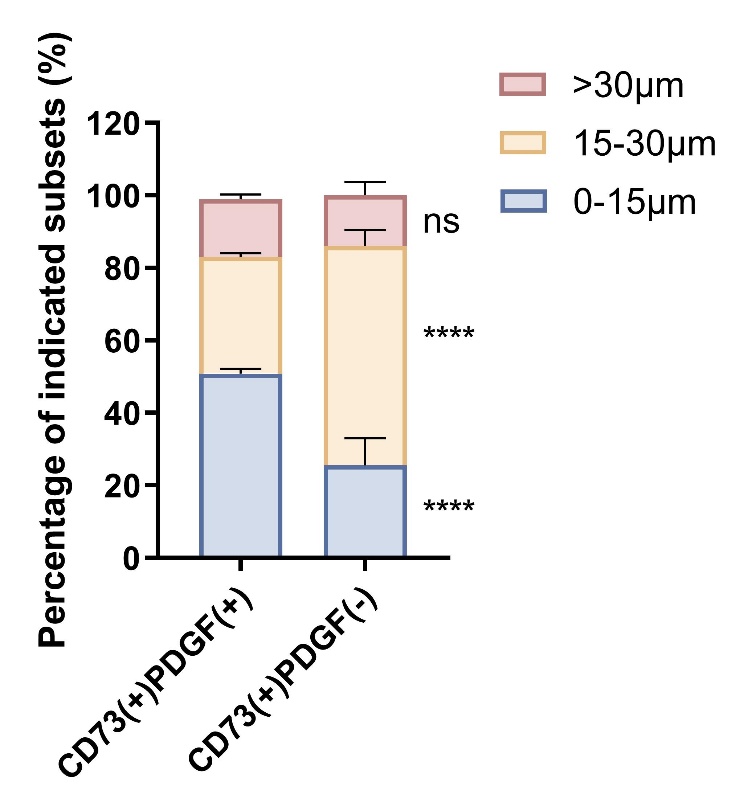


**Supplementary Figure 1.** **Quantitative analysis of PDGF (+) and PDGF (-) cell distribution**. The distance from each cell to the nearest blood vessel (identified by CD31 positivity) was systematically measured, followed by the calculation of cellular proportions within specified distance intervals. Statistical analysis revealed a significant spatial distribution difference, with the proportion of PDGF (+) cells within a 15-μm radius of blood vessels being approximately twice that of PDGF (-) cells (n=10,p < 0.0001). This spatial distribution pattern suggests a potential biological preference of PDGF (+) cells for perivascular localization.

## Supplementary Figure 2


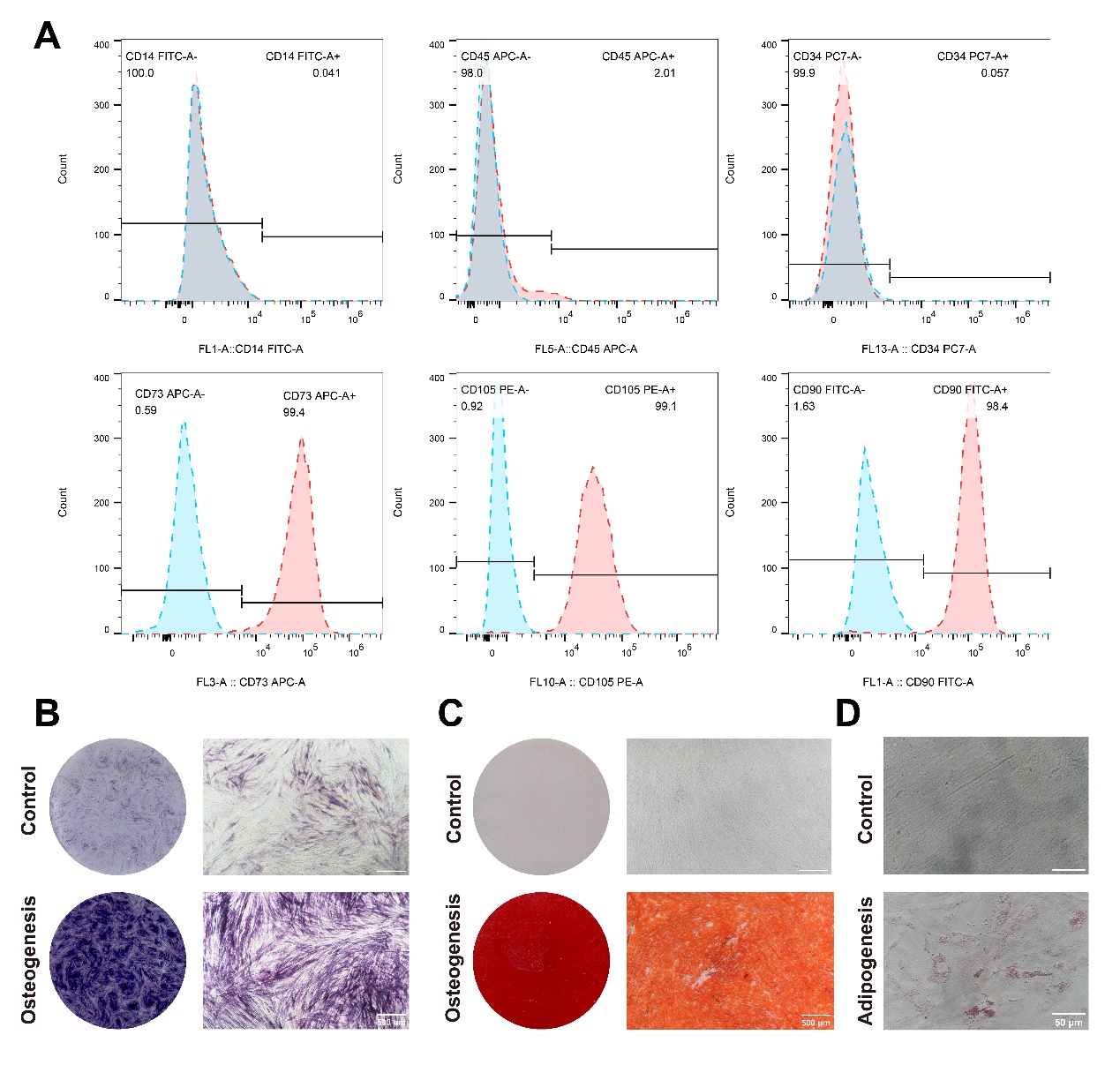


**Supplementary Figure 2 Validation of DPSC through FACS and differentiation assays.** (A)Flow cytometry results showed that the CD73, CD90 and CD105 were highly expressed in harvested cells, whereas these cells did not express the CD14, CD45, and CD31.(n=3) (B Alkaline phosphatase staining. Scale bar = 500 μm. (C Alizarin Red S staining. Scale bar = 500 μm .(D Oil Red O staining. Scale bar = 50μm.
